# Author Correction: Pharmacophore hybridisation and nanoscale assembly to discover self-delivering lysosomotropic new-chemical entities for cancer therapy

**DOI:** 10.1038/s41467-021-22419-2

**Published:** 2021-03-25

**Authors:** Zhao Ma, Jin Li, Kai Lin, Mythili Ramachandran, Dalin Zhang, Megan Showalter, Cristabelle De Souza, Aaron Lindstrom, Lucas N. Solano, Bei Jia, Shiro Urayama, Yuyou Duan, Oliver Fiehn, Tzu-yin Lin, Minyong Li, Yuanpei Li

**Affiliations:** 1 Department of Biochemistry and Molecular Medicine, UC Davis Comprehensive Cancer Center, University of California Davis, Sacramento, CA 95817 USA; 2 Department of Medicinal Chemistry, Key Laboratory of Chemical Biology (MOE), School of Pharmacy, Cheeloo College of Medicine, Shandong University, Jinan, 250012 Shandong China; 3 West Coast Metabolomics Center, University of California Davis, Davis, CA 95616 USA; 4 Division of Gastroenterology and Hepatology, Department of Internal Medicine, University of California Davis, Sacramento, CA 95817 USA; 5 Institutes for Life Sciences, School of Medicine, South China University of Technology, Guangzhou, 510006 Guangdong China; 6 Division of Hematology and Oncology, Department of Internal Medicine, University of California Davis, Sacramento, CA 95817 USA; 7 State Key Laboratory of Microbial Technology, Shandong University, Jinan, 250100 Shandong China

**Keywords:** Drug development, Drug delivery, Drug discovery and development, Drug delivery, Nanoparticles

Correction to: *Nature Communications* 10.1038/s41467-020-18399-4, published online 15 September 2020.

The original version of this Article omitted the following from the Acknowledgements:

L.N.S. was supported by the Training Grant in Oncogenic Signals and Chromosome Biology T32 CA108459.

This has now been corrected in both the PDF and HTML versions of the Article.

