# Supplementary material for: Pharmacophore hybridisation and nanoscale assembly to discover self-delivering lysosomotropic new-chemical entities for cancer therapy
Source: Nat Commun. 2020 Sep 15;11:4615. doi: 10.1038/s41467-020-18399-4 (PMC7493904; doi:10.1038/s41467-020-18399-4)
Supplement: Supplementary file 1 — Supplementary Information [file 41467_2020_18399_MOESM1_ESM.pdf]

Supplementary Information

Pharmacophore hybridization and nanoscale assembly to discover self-delivering lysosomotropic new chemical entities for cancer therapy

Ma *et al.*

Materials and instruments. Chemicals like Diethylenetriamine, Dodecyl aldehyde, fatty alcohol, pyridinium dichromate, sodium cyanoborohydride, ammonium hydroxide solution, deuterated solvents, anhydrous solvents, and Z-Arg-Arg-AMC were purchased from Millipore-Sigma (MO, USA). 4,7-Dichloroquinoline, anhydrous salt sulfate, and the bulk of solvents were purchased from Fisher Scientific (MA, USA). All solvents were used directly without further purification. Water used in all experiments was purified with a Mill-Q filtration system. Other reagents or drugs were purchased as indicated: tridecanal (Alfa Aesar), HCQ (Specturm), Lys05 (MedchemExpress), Bortezomib (eNovation chemical), DiD perchlorate and β-lapachone (Tocris Bioscience), JQ1 and Napabucasin (ApExBIO), rapamycin, paclitaxel and vinblastine (LC Laboratory), CN38 (Acros Organics), Etoposide (AdipoGen), Lenalidomide (Matrix Scientific), Napabucasin (ApExBIO) and Apoptozole (Selleck). Lysosome enrichment kit, LysoTracker (Red & Green), acridine orange, Dextran-Alexa Fluor 488, Premo™ Autophagy Sensor LC3B-GFP were bought from Thermo Fisher (MA, USA). The SensoLyte® homogeneous AMC caspase-3/7 assay kit and FITC-Annexin V/PI Apoptosis kit were bought from AnaSpec (CA, USA) and Biolegend (CA, USA), respectively. The compounds were characterized by a 600 MHz NMR spectrometer (Bruker, German) for NMR spectra and an LTQ-Orbitrap XL Hybrid ion trap mass spectrometer (Thermo Fisher, MA, USA) for ESI-HRMS spectra. Cell imaging studies were performed by a fluorescence microscope (Olympus, Tokyo, Japan) or a LSM800 confocal microscope (Carl Zeiss, Oberkochen, Germany). The absorbance and fluorescence intensity were determined with a SpectraMax M2 microplate reader (Molecular Devices, CA, USA). Western Blot was developed by a Power Pac 200 electrophoresis apparatus (Bio-Rad, CA, USA). The studies, including WB imaging, *in vivo,* and *ex vivo* fluorescence imaging, were performed on a ChemiDocTM MP imaging system (Bio-Rad, CA, USA). DLS experiments were done with a Zetasizer Nano ZS (Malvern Instruments, Worcestershire, UK). TEM was performed on a Talos L120C TEM (FEI, OR, USA) with 80 kV acceleration voltage. Apoptosis assay was carried out by using a BD FACSCanto II flow cytometer (BD Biosciences, NJ, USA). Isolation of cancer stem cells was conducted by a BD FACSAria II Cell Sorter (BD Biosciences, NJ, USA). The Matrigel for 3D culture (Cat# 354230) and xenograft model establishment (Cat# 354234) were both purchased from Corning (NY, USA). LC3B antibody (1:1000, Catalog: #2775), SQSTM1/p62 antibody

(1:1000, Catalog: #39749) and β-actin antibody (1:1000, Catalog: #4970) were purchased from Cell Signaling, and Pacific Blue anti-CD44 antibody (5 μL per million cells in 100 μL staining volume, Catalog: #338823); APC anti-CD326 (EpCAM) antibody (5 μL per million cells in 100 μL staining volume, Catalog: #324207); PE/Cy7 anti-CD24 antibody (5 μL per million cells in 100 μL staining volume, Catalog: #311119) were obtained from Biolegend.

Synthesis of MSDH. MSDH was synthesized according to a published literature (*Bioorg. Med. Chem. Lett*. **1995**, 5, 893−898). ^{1}H NMR (600 MHz, $CDCl_3$): δ 7.40 (s, 1 H), 3.63 (m, 1 H), 3.58 (m, 2 H), 3.70 (s, 3 H), 3.26 (m, 2 H), 1.74 (s, 2 H), 1.51 (m, 2 H), 1.29 (m, 18 H), 0.89 (t, 3 H, *J*=7.2 Hz). ESI-HRMS: m/z $[M+H]^+$ calcd for $C_{16}H_{35}N_2O_2^+$ 287.2693, found 287.2690.

Synthesis of BAQ. 4,7-dichloroquinoline (1.2 g, 6.00 mmol) in a 10 mL flask was maintained at 80°C for 2 h without stirring, followed by adding diethylenetriamine (0.22 mL, 2.00 mmol). The reaction solution was stirred at 130°C for 6 h. The residue was taken up with 30 mL methanol to afford white solid as the BAQ compound. Yield: 470 mg, 55%. ^{1}H NMR (600 MHz, DMSO-d_6): δ 8.38 (d, 2 H, *J*=6.0 Hz), 8.23 (d, 2 H, *J*=10.8 Hz), 7.78 (d, 2 H, *J*=1.8 Hz), 7.42 (dd, 2 H, J_1=10.8 Hz, J_2=2.4 Hz), 7.25 (s, 1 H), 6.51 (d, 2 H, *J*=6.6 Hz), 3.40 (t, 4 H, *J*=7.2 Hz), 2.94 (t, 4 H, *J*=7.8 Hz). ESI-HRMS: m/z $[M+H]^+$ calcd for $C_{22}H_{22}Cl_2N_5^+$ 426.1247, found 426.1243.

General synthetic method of BAQ12-18. To the solution of BAQ (426 mg, 1.0 mmol) in 30 mL anhydrous methanol and 10 mL anhydrous dichloromethane was added the corresponding aldehyde (2 mmol) and acetic acid (20 μL) and then was stirred for 20 min at room temperature, followed by adding sodium cyanoborohydride (126 mg, 2 mmol). The mixture was stirred for 12 h and was diluted by chloroform (100 mL). The organic phase was collected, washed with water, and dried by anhydrous sodium sulfate overnight. The crude product was purified via silica gel chromatography with the eluent containing 0.1% triethylamine (dichloromethane: methanol=30:1-10:1) to afford the corresponding compound.

BAQ12. Yield: 320 mg, 53.8%. ^{1}H NMR (600 MHz, CD_3OD): δ 8.27 (d, 2 H, *J*=5.4 Hz), 7.67 (d, 2 H, *J*=2.4 Hz), 7.55 (d, 2 H, *J*=9.0 Hz), 6.95 (dd, 2 H, J_1=9.0 Hz, J_2=1.8 Hz), 6.46 (d, 2 H, *J*=5.4 Hz), 3.41 (t, 4 H, *J*=6.0 Hz), 2.90 (t, 4 H, *J*=6.0 Hz), 2.66 (t, 2 H, *J*=6.6 Hz), 1.55 (m, 2 H), 1.33 (m, 20 H), 0.91 (t, 3 H, *J*=7.2 Hz). ^{13}C NMR (150 MHz, CD_3OD): δ 150.9, 150.8, 147.9, 134.8, 126.3, 124.4, 121.9, 117.0, 98.4, 54.1,

51.9, 40.3, 31.6, 29.5, 29.5, 29.4, 29.4, 29.1, 27.4, 27.3, 22.4, 13.1. ESI-HRMS: m/z $[M+H]^+$ calcd for $C_{34}H_{46}Cl_2N_5^+$ 594.3125, found 594.3134.

BAQ13. Yield: 350 mg, 57.5%. ^{1}H NMR (600 MHz, CD_3OD): δ 8.27 (d, 2 H, *J*=5.4 Hz), 7.67 (d, 2 H, *J*=1.8 Hz), 7.56 (d, 2 H, *J*=9.0 Hz), 6.96 (dd, 2 H, J_1=9.0 Hz, J_2=2.4 Hz), 6.46 (d, 2 H, J=5.4 Hz), 3.42 (t, 4 H, *J*=6.0 Hz), 2.90 (t, 4 H, *J*=6.0 Hz), 2.66 (t, 2 H, *J*=7.2 Hz), 1.56 (m, 2 H), 1.32 (m, 23 H), 0.92 (t, 3 H, *J*=7.2 Hz). ^{13}CNMR (150 MHz, CD_3OD): δ 151.0, 150.7, 147.8, 134.8, 126.1, 124.5, 121.9, 117.0, 98.4, 54.6, 51.9, 40.3, 31.7, 29.5, 29.5, 29.5, 29.4, 29.1, 27.4, 27.3, 22.3, 13.1. ESI-HRMS: m/z $[M+H]^+$ calcd for $C_{35}H_{48}Cl_2N_5^+$ 608.3281, found 608.3274.

BAQ14. Yield: 295 mg, 47.4%. ^{1}H NMR (600 MHz, CD_3OD): δ 8.56 (d, 2 H, *J*=9.0 Hz), 8.48 (d, 2 H, *J*=4.8 Hz), 7.86 (d, 2 H, *J*=1.2 Hz), 7.60 (dd, 2 H, J_1=9.0 Hz, J_2=1.2 Hz), 7.06 (d, 2 H, *J*=5.4 Hz), 4.16 (s, 4 H), 3.82 (s, 4 H), 3.48 (s, 2 H), 1.92 (s, 2 H), 1.44 (s, 2 H), 1.35 (m, 23 H), 0.93 (t, 3 H, *J*=6.6 Hz). ^{13}C NMR (150 MHz, CD_3OD): δ 155.9, 143.0, 139.8, 138.2, 127.4, 125.3, 118.7, 115.5, 98.9, 54.6, 51.1, 38.3, 31.5, 29.2, 29.2, 29.2, 29.2, 29.1, 29.0, 28.9, 28.7, 26.1, 23.0, 22.2, 12.9. ESI-HRMS: m/z $[M+H]^+$ calcd for $C_{36}H_{50}Cl_2N_5^+$ 622.3438, found 622.3505.

BAQ15. Yield: 290 mg, 45.5%. ^{1}H NMR (600 MHz, CD_3OD): δ 8.56 (d, 2 H, *J*=9.0 Hz), 8.48 (d, 2 H, *J*=6.0 Hz), 7.86 (d, 2 H, *J*=1.2 Hz), 7.59 (dd, 2 H, J_1=9.0 Hz, J_2=1.2 Hz), 7.07 (d, 2 H, *J*=6.0 Hz), 4.16 (s, 4 H), 3.82 (s, 4 H), 3.48 (s, 2 H), 1.92 (s, 2 H), 1.44 (s, 2 H), 1.35 (m, 25 H), 0.93 (t, 3 H, *J*=6.6 Hz). ^{13}C NMR (150 MHz, CD_3OD): δ 155.8, 143.0, 139.7, 138.1, 127.3, 125.3, 118.7, 115.4, 98.9, 54.5, 51.0, 38.2, 31.5, 29.2, 29.2, 29.2, 29.1, 29.0, 28.9, 28.7, 26.1, 23.0, 22.2, 12.9. ESI-HRMS: m/z $[M+H]^+$ calcd for $C_{37}H_{52}Cl_2N_5^+$ 636.3594, found 636.3661.

BAQ16. Yield: 280 mg, 43.0%. ^{1}H NMR (600 MHz, CD_3OD): δ 8.56 (d, 2 H, *J*=9.0 Hz), 8.48 (d, 2 H, *J*=6.6 Hz), 7.86 (d, 2 H, *J*=1.8 Hz), 7.59 (dd, 2 H, J_1=9.0 Hz, J_2=1.8 Hz), 7.07 (d, 2 H, *J*=7.2 Hz), 4.17 (m, 4 H), 3.84 (m, 4 H), 3.50 (t, 2 H, *J*=7.8 Hz), 1.92 (m, 2 H), 1.44 (m, 2 H), 1.30 (m, 27 H), 0.93 (t, 3 H, *J*=7.2 Hz). ^{13}C NMR (150 MHz, CD_3OD): δ 155.9, 143.0, 139.7, 138.1, 127.3, 125.2, 118.7, 115.4, 98.8, 54.5, 51.0, 38.2, 31.5, 29.2, 29.2, 29.2, 29.1, 28.9, 28.9, 28.7, 26.1, 23.0, 22.1, 12.9. ESI-HRMS: m/z $[M+H]^+$ calcd for $C_{38}H_{54}Cl_2N_5^+$ 650.3751, found 650.3774.

BAQ18. Yield: 290 mg, 42.7%. ^{1}H NMR (600 MHz, CD_3OD): δ 8.56 (d, 2 H, *J*=9.0 Hz), 8.48 (d, 2 H, *J*=5.4 Hz), 7.86 (s, 2 H, *J*=1.8 Hz), 7.56 (d, 2 H, J_1=8.4 Hz), 7.06 (d, 2 H, *J*=6.6 Hz), 4.16 (s, 4 H), 3.82 (m, 4 H),

3.48 (s, 2 H), 1.91 (s, 2 H), 1.43 (m, 2 H), 1.30 (m, 29 H), 0.93 (s, 3 H). ^{13}C NMR (150 MHz, CD_3OD): δ 155.9, 143.0, 139.8, 138.2, 127.4, 125.2, 118.7, 115.5, 98.8, 54.5, 51.1, 38.2, 31.5, 29.2, 29.1, 28.9, 28.9, 28.7, 26.1, 23.0, 22.2, 12.9. ESI-HRMS: m/z $[M+H]^+$ calcd for $C_{38}H_{54}Cl_2N_5^+$ 650.3751, found 650.3774. ESI-HRMS: m/z $[M+H]^+$ calcd for $C_{40}H_{56}Cl_2N_5^+$ 678.4064, found 678.4069.

Cell line and cell culture. The human pancreatic cancer cell lines (MIA PaCa-2, BXPC3, and PANC-1)were originally purchased from ATCC and were kindly provided by Dr. Shiro Urayama's Lab. HT29, HCT116, H460, MCF7, NIH/3T3, and IMR-90 cell lines were purchased from ATCC. Bone marrow cells were collected from the leg bone marrows of FVB/N mice. All the cells were cultured at 37°C in a humidified atmosphere of 5% CO_2/95% air using the corresponding medium supplemented with 10% fetal bovine serum, 100 μg mL^{-1} penicillin and 100 units mL^{-1} of streptomycin according to ATCC protocol. All the cell lines have been tested for mycoplasma contamination routinely.

Animal feeding. All animal experiments were conducted in accordance with the protocol (#20265) approved by the Institutional Animal Care and Use Committee at the University of California, Davis. Female mice (4-6 week) including BALB/c nude mice (Envigo), NRG mice (Jackson Laboratory), and FVB/N mice (Charles River) were purchased and group-housed under standard conditions (22 ± 1°C, humidity 50-60%, 12 h light/12 h dark cycle, free access to food and water).

Establishment of patient-derived pancreatic cancer stem cells (PCSCs). The pancreatic patient tissue was donated by Dr. Shiro Urayama's Lab from UC Davis Medical Center. Patient consent was obtained for the use of "Remnant Clinical Biospecimens" in accordance with the Institutional Review Board (UC Davis IRB Protocol #244896). The patient tumour tissue was harvested using Collagenase IV and Dispase (Stem Cell Technologies, Vancouver, Canada) and strained through 70 μm filters. By labeled with the following antibodies (Biolegend, CA, USA): anti-CD44 (IM7, Cat: #338823), anti-CD326 (9C4, Cat: #324207) and anti-CD24 (ML5, Cat: #311119), the cells were isolated using a BD FACSAria II Cell Sorter (**Supplementary Fig. 10**). Purified PCSCs were collected and maintained in Essential 8 Flex Medium (Thermo Fischer) and trypsinized using Gentle Cell Dissociation Reagent (Stem Cell Technologies, Vancouver, Canada). In tumour sphere-formation assay, the cold single cell suspension of PCSCs in Essential 8 Flex Medium was mixed with cold Matrigel (1:1, volume ratio), followed by slowly and uniformly dropping them (100 μL) into well center on a 24-well plate (5,000 cells per well). The Matrigel was allowed

to solidify in a humidified incubator at 37°C for 45-60 min, and the warm media (500 μL) was added into each well. The tumour sphere was allowed to be formed in two weeks. For tumourigenicity *in vivo*, PCSCs were counted and resuspended into a mixture of PBS and Matrigel (1:1) and subsequently injected subcutaneously into the flanks of NRG mice.

***In vivo* toxicity studies.** The toxicity of BAQ NPs was investigated on female FVB/N mice via iv injection. Mice were administrated with various concentrations (10 mg kg^{-1}, 20 mg kg^{-1} or 40 mg kg^{-1}) of Lys05, Liposomes@Lys05, BAQ12 NPs, and BAQ13 NPs every two days. The status of mice was monitored every day and their body weight was recorded every two days. Blood samples were collected and sent to the UCD Comparative Pathology Laboratory for tests of complete blood count (CBC) and serum chemistry.

***In vivo* pharmacokinetic study.** The jugular vein of female Sprague-Dawley rats (200-250g) was implanted with a catheter for drug injection and blood collection (Harland, Indianapolis, IN, USA). Rats (n = 3) were injected with free DiD, BAQ12 NPs@DiD (10:1, mass ratio) and BAQ13 NPs@DiD (10:1, mass ratio), respectively, which contained an equivalent dose of DiD (0.5 mg kg^{-1}). Blood samples were collected at the indicated time points and then were centrifuged to obtain the plasma. The plasma was diluted with DMSO (1:100), and the fluorescence intensity (λ_{Ex}=595 nm, λ_{Em}=665 nm) was measured by a microplate reader (SpectraMax M2).

***In/ex vivo* biodistribution.** Nude mice bearing the HT29 tumours were subjected to iv administration of BAQ13 NPs@DiD (10:1, mass ratio) at a dose of 1.0 mg kg^{-1} DiD. *In vivo* imaging studies were performed at the corresponding time point. Organs (brain, heart, lung, liver, spleen, kidney, intestines, and muscle) and tumours were collected from mice for *ex vivo* imaging. Biodistribution of BAQ13 NPs@ NAPA+DiD (10/2.5/1.0 mg kg^{-1}, iv) was studied on NRG mice bearing PCSC tumours. Both *in vivo* and *ex vivo* imaging studies were performed as above.

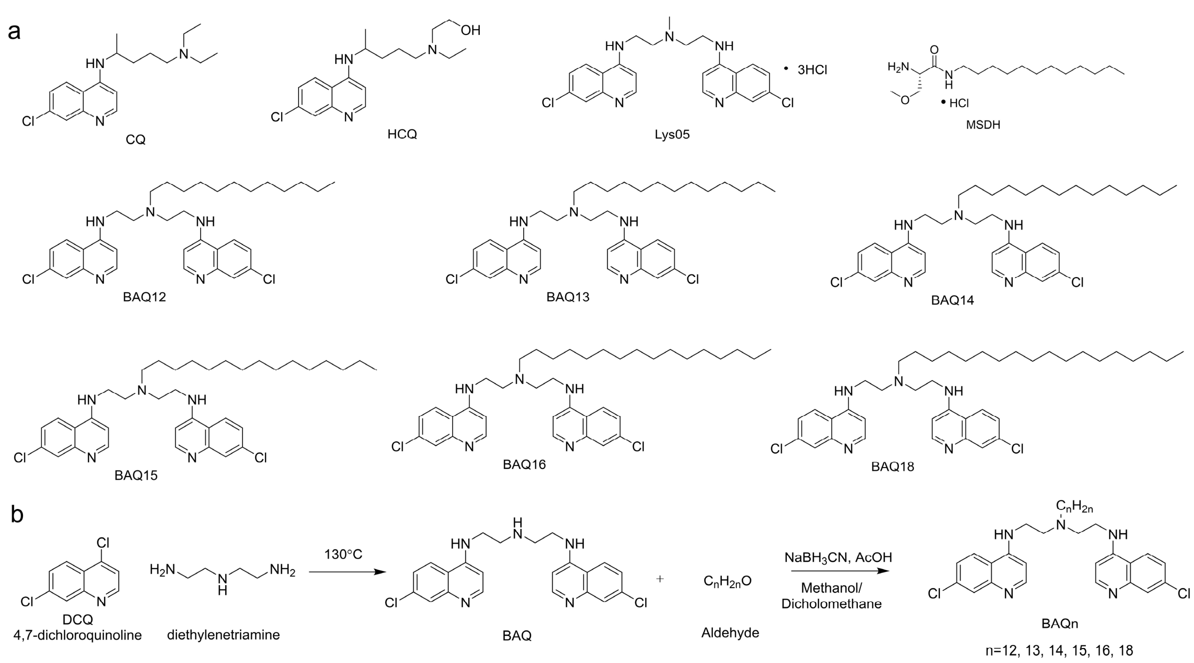

Supplementary Fig. 1 Compound list and synthesis method. (**a**) Chemical structures of compounds involved in this work. (**b**) Synthetic route of BAQ12-BAQ18.

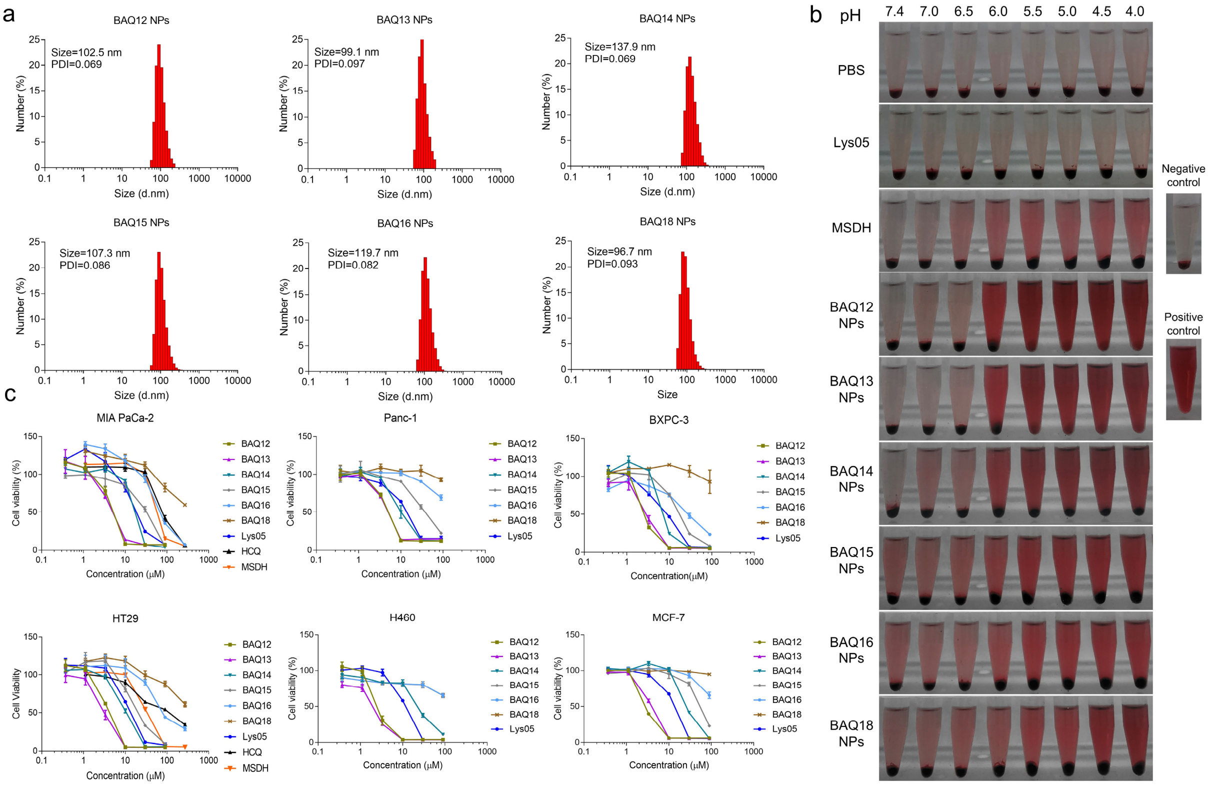

Supplementary Fig. 2 *In vitro* evaluation of BAQ NPs. (**a**) Size distribution. (**b**) Observation of pH-dependent haemolytic effect; Red blood cells were treated as indicated (50 μM, 4 h). (**c**) Viability curves of various cell lines that were exposed to different compounds for 24 h, respectively. Data are presented as mean values ± SD; *n* = 3 independent experiments.

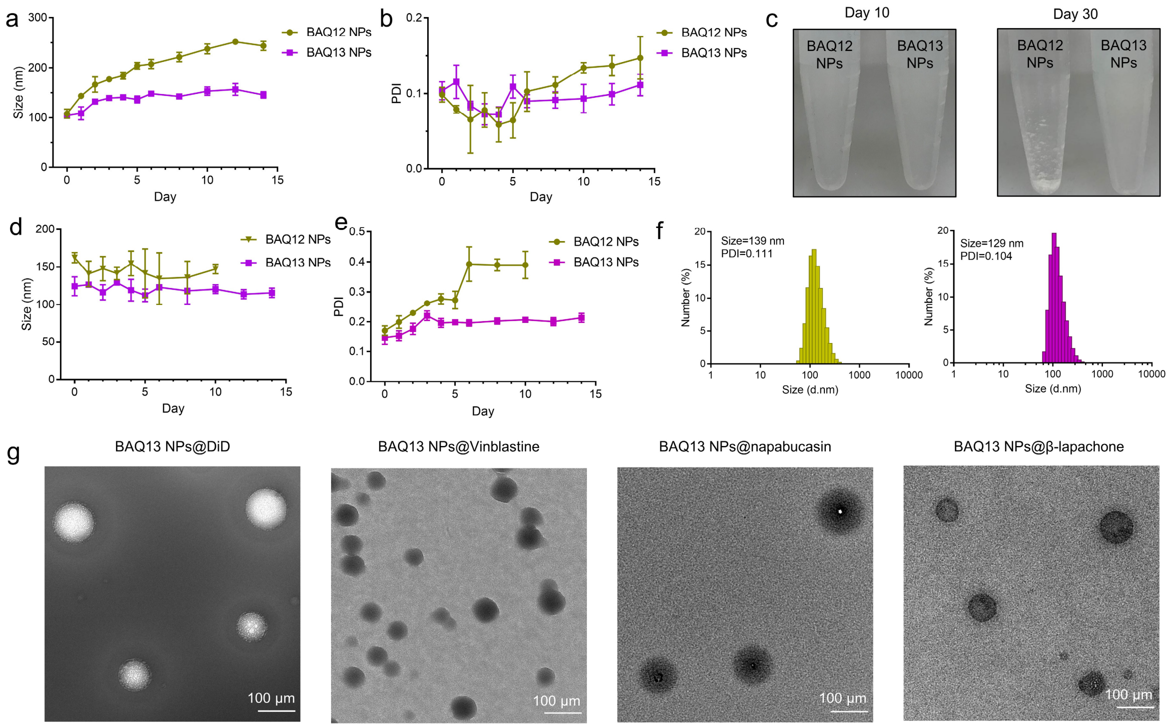

Supplementary Fig. 3 Stability measurements and TEM characterization of BAQ NPs. (**a**, **b**) Dynamic Light Scattering (DLS) measurements of BAQ12 NPs and BAQ13 NPs in neutral condition; Data are presented as mean values ± SD; *n* = 3. (**c**) Whole appearance of BAQ12 NPs and BAQ13 NPs at Day 10 and 30. (**d, e**) DLS measurements of BAQ12 NPs and BAQ13 NPs in presence of 10% FBS; Data are presented as mean values ± SD; *n* = 3. (**f**) Size distribution of BAQ12 NPs (left) and BAQ13 NPs (right) at 24 h post-incubation with 0.5 mM BSA. (**g**) Representative TEM images of BAQ13 NPs that loads different agents; Experiments were repeated three times independently.

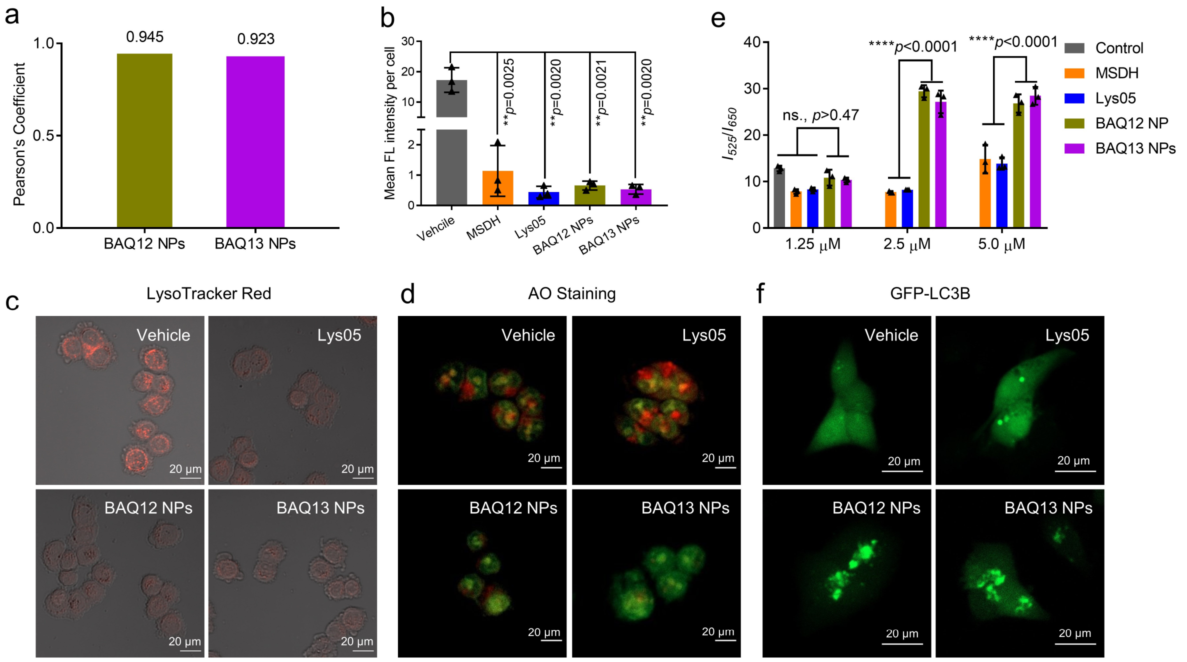

Supplementary Fig. 4 Effect of BAQ NPs on lysosomes and autophagy on cell level. (**a**) Pearson correlation coefficients for colocalization analysis of Fig. 3a. (**b**) Fluorescence quantification of LysoTracker Green in MIA PaCa-2 cells that were treated as indicated; Data are presented as mean values ± SD; *n* = 3 independent experiments; Statistical significance was calculated by the two-tailed Student's t-test; ***p*<0.01. (**c**) HT29 cells were treated as indicated (10 μM, 2 h) and then were stained with LysoTracker Red; Experiments were repeated three times independently. (**d**) AO staining of HT29 cells treated as indicated (5 μM, 12 h); Experiments were repeated three times independently. (**e**) Ratio of fluorescence intensity at 525 nm and 650 nm in MIA PaCa-2 cells that were treated as indicated for 12 h; Data are presented as mean values ± SD; *n* = 3 independent experiments; Statistical significance was calculated by one-way ANOVA with the Tukey's multiple comparison test; ns., not significant; *****p*<0.0001. (**f**) Representative LC3B-GFP images in cells within the corresponding treatments (5 μM, 4 h); Experiments were repeated three times independently.

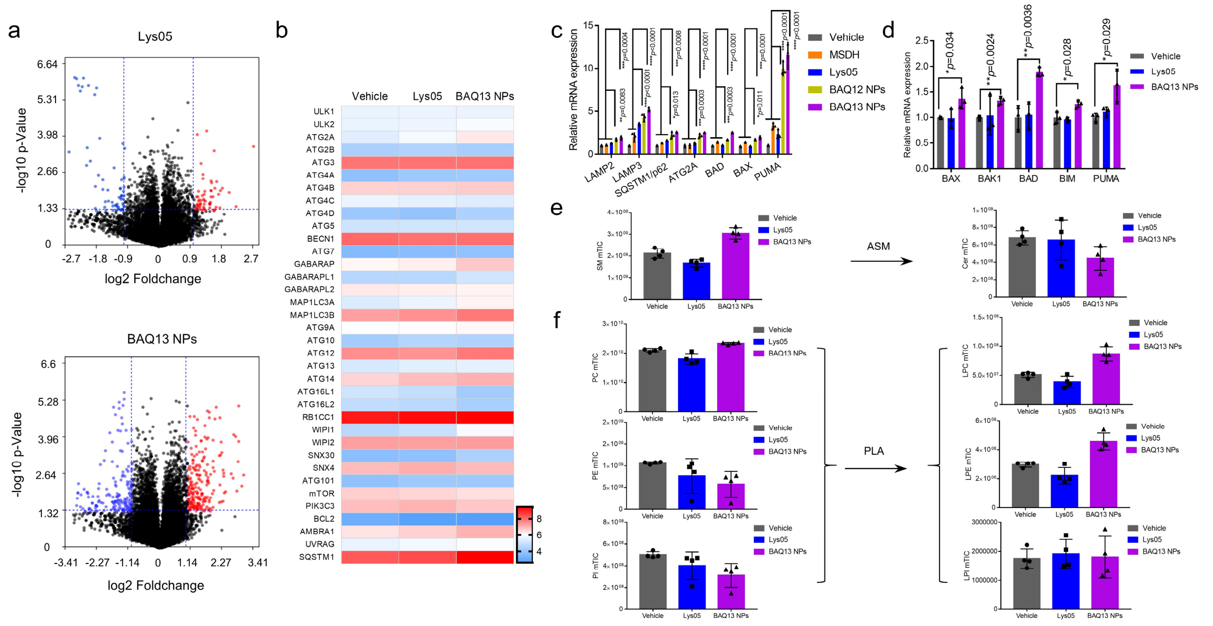

Supplementary Fig. 5 Effect of BAQ NPs on gene expression and lipid metabolism. (**a**) Volcano plots from RNA-seq showing differentially expressed genes in MIA PaCa-2 cells induced by Lys05 (bottom) and BAQ13 NPs (upper). (**b**) Change of autophagy-associated genes according to the RNA-seq results. (**c**) qPCR analysis of representative autophagy- and apoptosis-associated genes of MIA PaCa-2 cells that were treated as indicated (5 μM, 24 h); Data are presented as mean values ± SD; *n* = 3; Statistical significance was calculated by one-way ANOVA with the Tukey's multiple comparison test; **p*<0.05; ***p*<0.01; ****p*<0.001; *****p*<0.0001. (**d**) Change of apoptosis-associated genes of MIA PaCa-2 cells that were treated as indicated (5 μM, 12 h); Data are presented as mean values ± SD; *n* = 3; Statistical significance was calculated by the two-tailed Student's t-test; **p*<0.05; ***p*<0.01. (**e**, **f**) Concentration alteration of acid sphingomyelinase (ASM) substrates (**e**) and phospholipase A (PLA) substrates (**f**) in MIA PaCa-2 cells with or without treatment (2.5 μM, 48 h); Data are presented as mean values ± SD; *n* = 4.

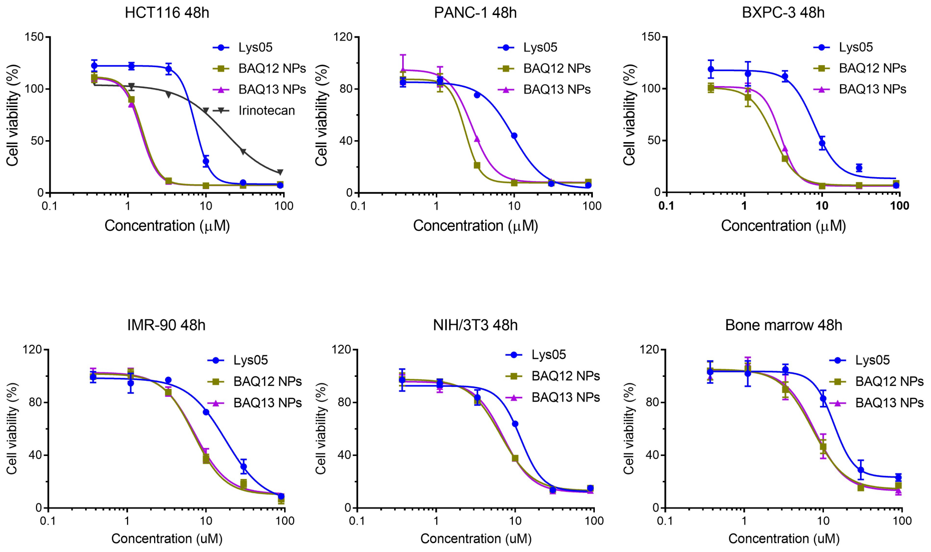

Supplementary Fig. 6 Viability curves of various cell lines that were treated as indicated for 48 h. Data are presented as mean values ± SD; *n* = 3 independent experiments.

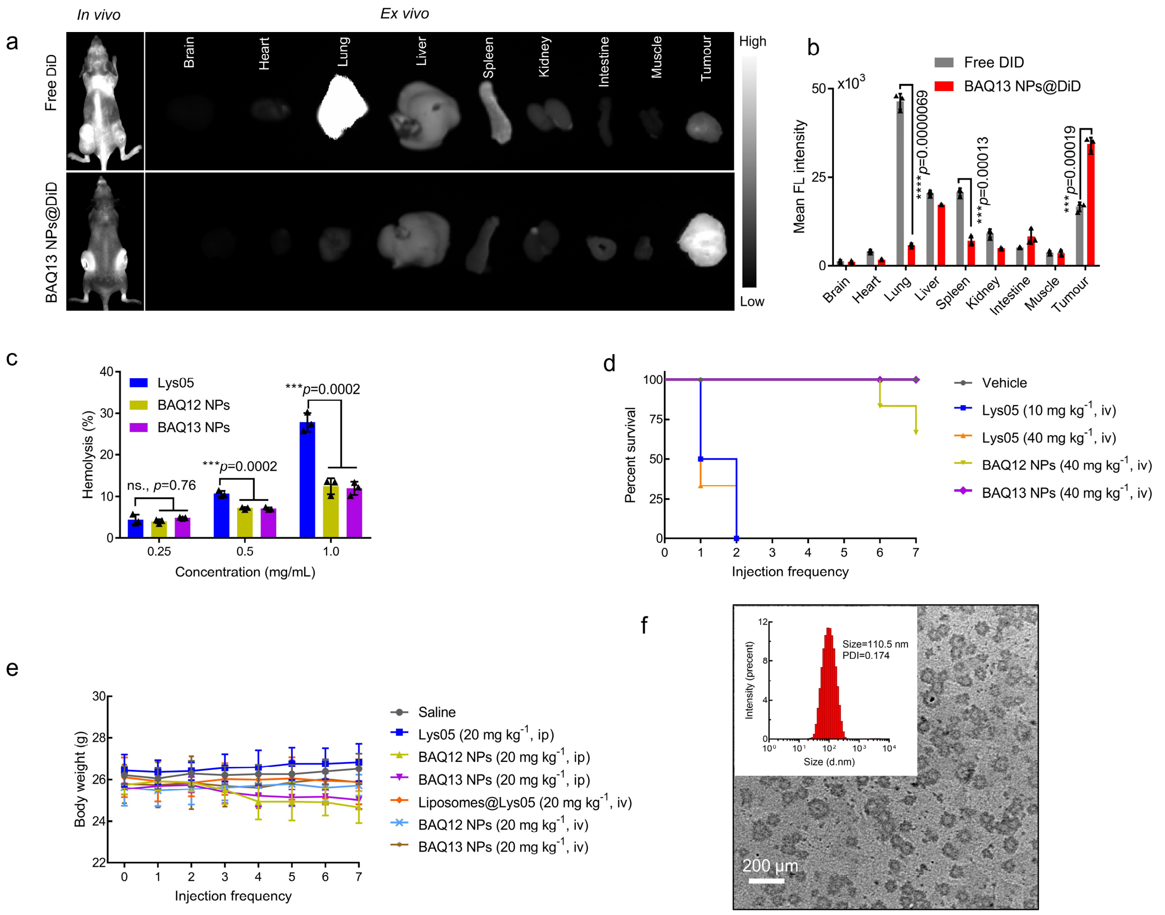

Supplementary Fig. 7 Biodistribution and toxicity studies of BAQ NPs. (**a**) *In vivo* and *ex vivo* imaging of mice bearing HT29 tumours after 12 h post-injection (i.v.) with free DiD (upper) and DiD-loaded BAQ13 NPs (lower), n = 3 mice per group. (**b**) Quantitative fluorescence intensity of organs in **a**; Data are presented as mean values ± SD; n = 3 mice per group; Data are presented as mean values ± SD; n = 3; Statistical significance was calculated by the two-tailed Student's t-test; ***p<0.001; ****p<0.0001. (**c**) Concentration-dependent haemolysis induced by the corresponding treatments in physiological pH; Data are presented as mean values ± SD; n = 3 independent nanoparticle samples; Statistical significance was calculated by one-way ANOVA with the Tukey's multiple comparison test; ns., not significant; ***p<0.001; ****p<0.0001. (**d**) Survival of FVB/n mice that were i.v. injected with the corresponding agents every two days; n = 6 mice per group. (**e**) Body weight of mice that were treated every two days as indicated; Data are presented as mean values ± SD; n = 4 mice per group. (**f**) The Dynamic Light Scattering (DLS) measurement and representative TEM image of liposomes@Lys05; Experiments were repeated three times independently.

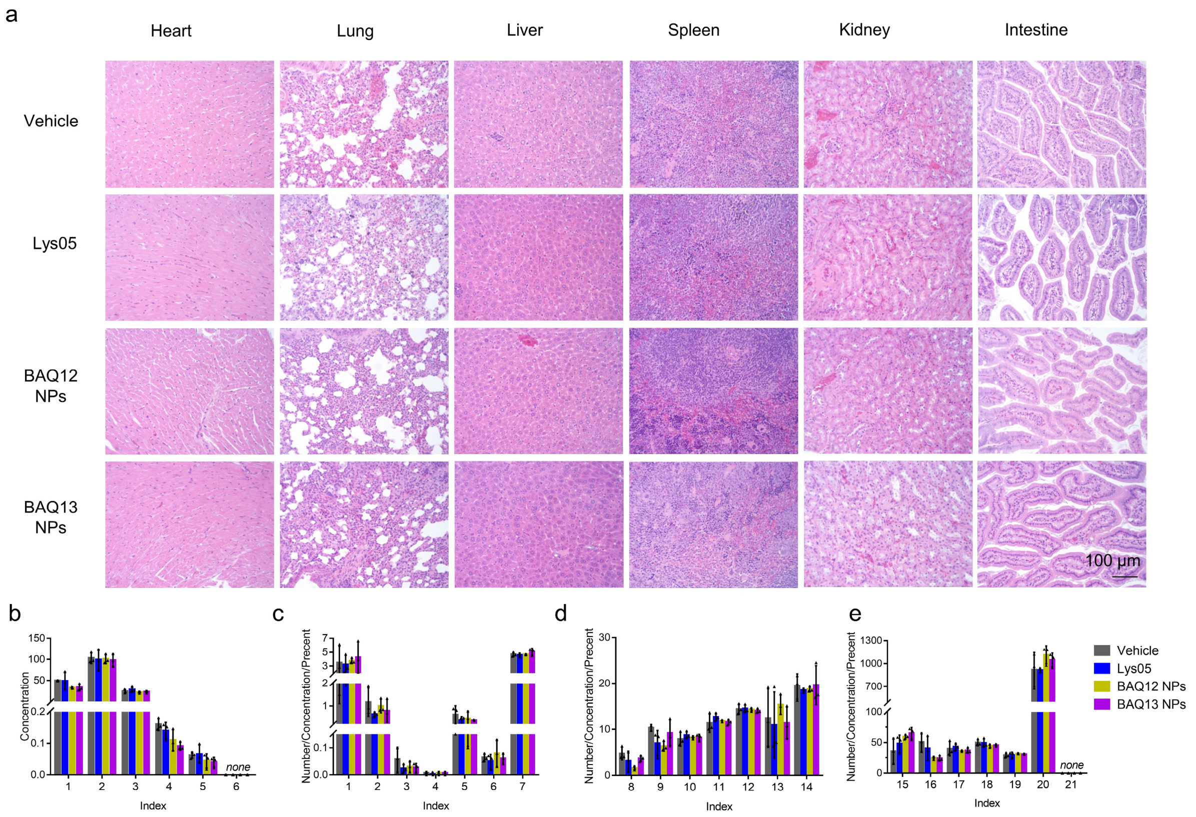

Supplementary Fig. 8 Analysis of tissue sections and haematology. (**a**) H&E analysis of major organs from mice that were treated with vehicle (Saline), Lys05 (20 mg kg^{-1}, ip), BAQ12 NP (20 mg kg^{-1}, iv), and BAQ13 NP (20 mg kg^{-1}, iv) for 24 days. The scale bar is 100 μm. Experiments were repeated three times independently. (**b**) The corresponding serum chemistry analysis of mice in **a**. 1. Alanine Transaminase U L^{-1}, 2. Aspartate Transaminase U L^{-1}, 3. Blood Urea Nitrogen mg dL^{-1}, 4, Creatinine mg dL^{-1}, 5. Total Bilirubin mg dL^{-1}, 6. Hemolysis. (**b**-**e**) The corresponding complete blood count (CBC) analysis of mice in **a**; Data are presented as mean values ± SD; n = 3 mice per group. 1. Absolute Neutrophil cells (k μL^{-1}), 2. Absolute Monocyte cells (k μL^{-1}), 3. Absolute Eosinophil cells (k μL^{-1}), 4. Absolute Basophil cells (k μL^{-1}), 5. Eosinophil %, 6. Basophil %, 7. MPV (fL), 8. Absolute Lymphocyte cells (k μL^{-1}), 9. WBC (k μL^{-1}), 10. RBC (M μL^{-1}), 11. Hemoglobin (g dL^{-1}), 12. MCH (pg), 13. Monocyte (%), 14. RDW (%), 15. Neutrophil %, 16. Lymphocyte %, 17. Hematocrit %, 18. MCV (fL), 19. MCHC (g dL^{-1}), 20. Platelets (k μL^{-1}), 21. Presence of clots.

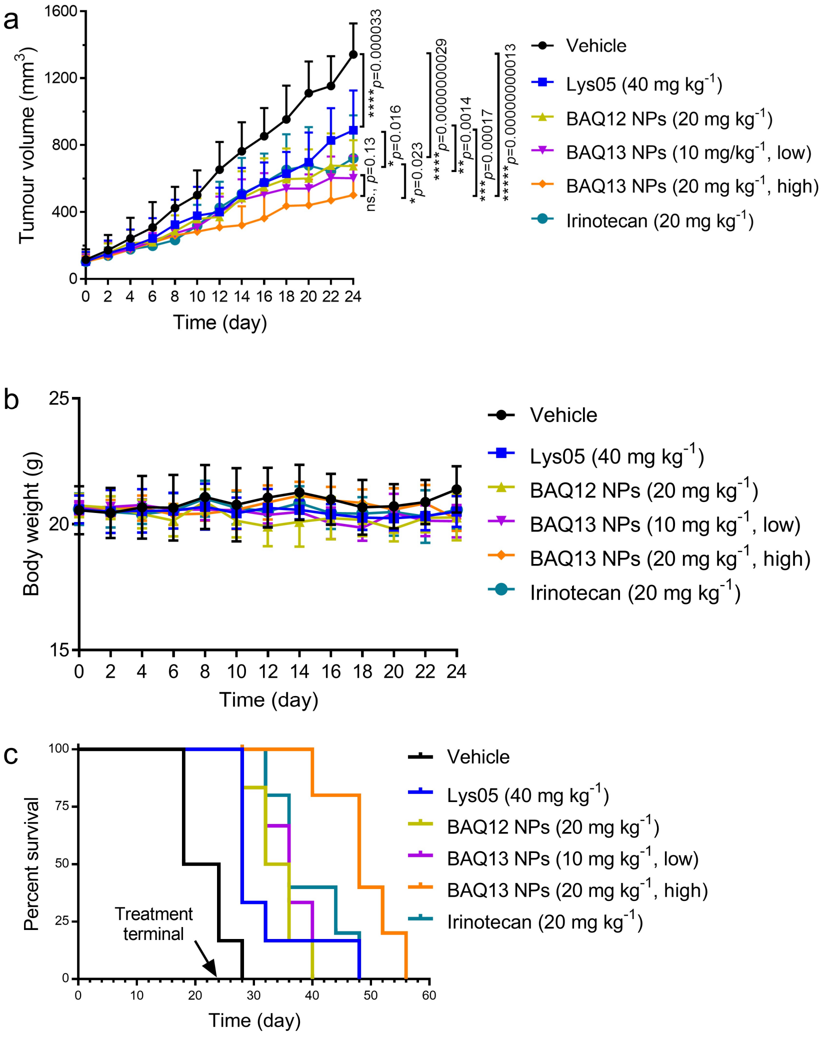

Supplementary Fig. 9 *In vivo* evaluation of BAQ NPs in HT29 mouse model. (**a**) Tumour growth curves in the subcutaneous HT29 xenograft model within intravenous administration every two days; Data are presented as mean values ± SD; n = 12 tumours per group. The statistical significance was calculated by the two-tailed Student's t-test; ns., not significant; *p<0.05; **p<0.01; ***p<0.001, ****p<0.0001. (**b**) Body weight of mice during the treatment; Data are presented as mean values ± SD; n = 6 mice per group. (**c**) Survival curves of mice; n = 6 mice per group. The treatment was terminated on the 24th day.

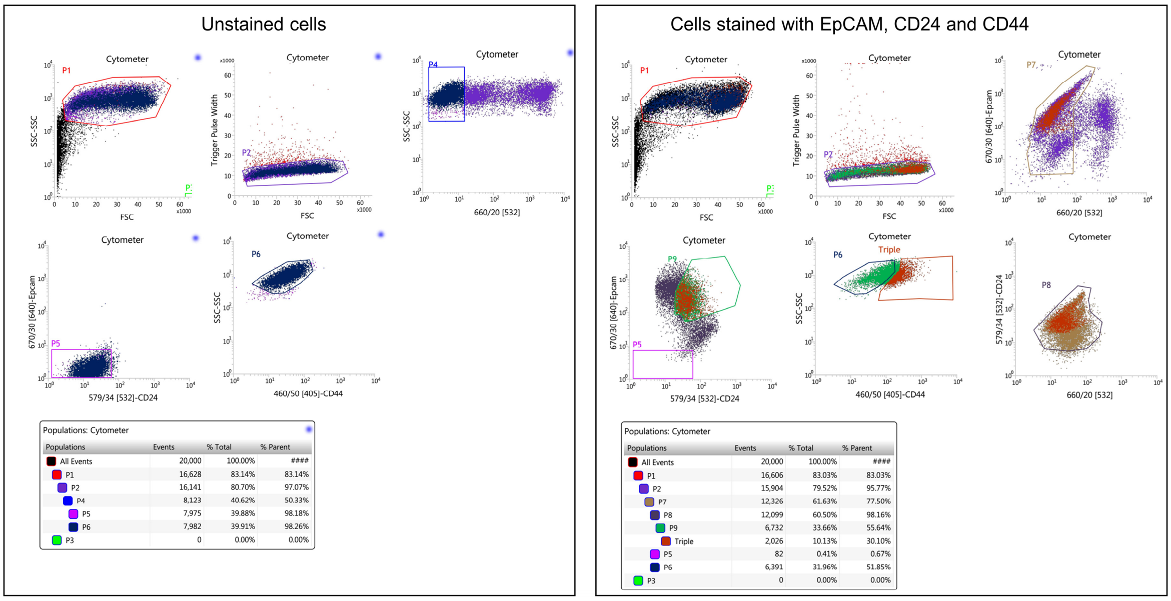

Supplementary Fig. 10 Gating strategy for isolation of pancreatic cancer stem cells (PCSCs).

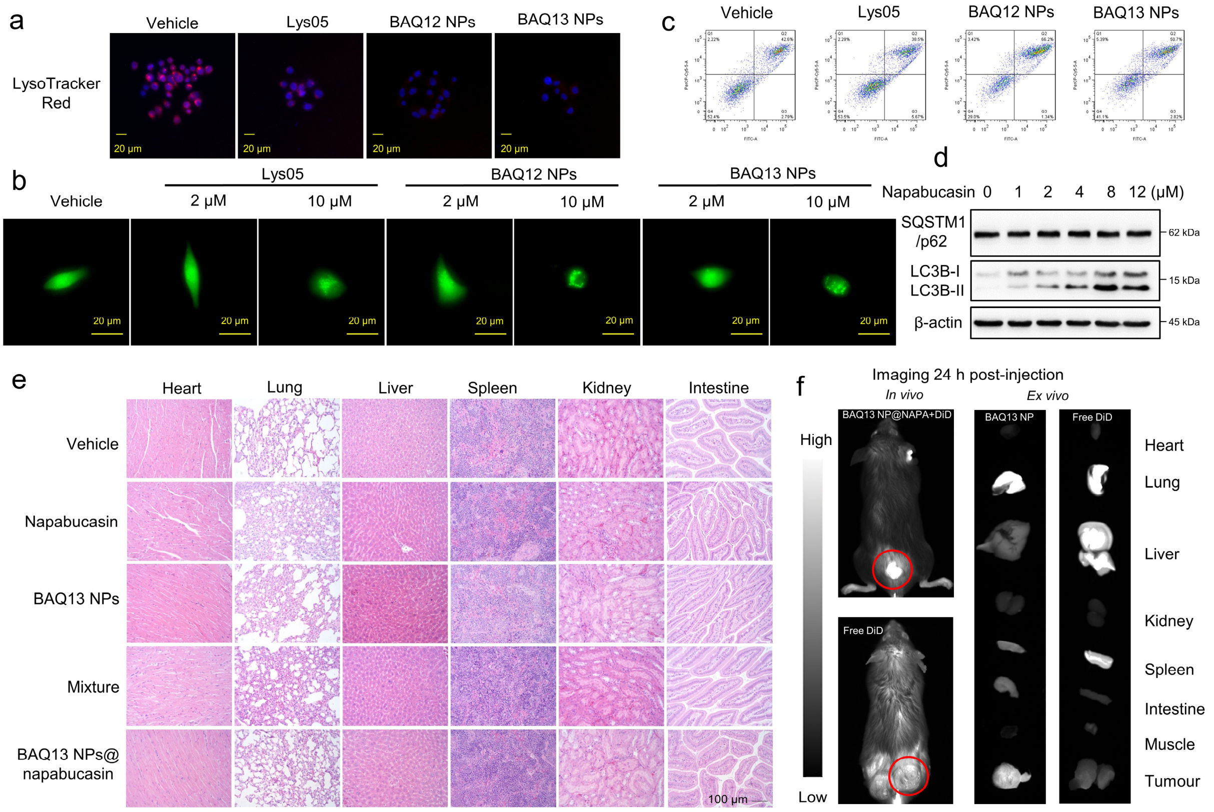

Supplementary Fig. 11 Evaluation of BAQ NPs in the model of pancreatic cancer stem cells (PCSCs). (**a**) Lysosomal deacidification assay. PCSCs were treated for 2 h (10 μM) and then were stained by LysoTracker Red; Experiments were repeated three times independently. (**b**) Representative images of LC3B-GFP-expressing PCSCs that were treated as indicated (5 μM, 4 h); Experiments were repeated three times independently. (**c**) Percentage of the apoptotic population of PCSCs within the indicated treatments (5 μM, 24 h). (**d**) Immunoblotting assay of PCSCs that were treated as indicated for 8 h; Experiments were repeated three times independently. (**e**) H&E analysis of major organs that were harvested at the end of treatments; Experiments were repeated three times independently. (**f**) Biodistribution of free DiD and BAQ13 NPs@napabucasin+DiD in mice after 24 h post-injection by tail vein; Red circles: tumours.

Supplementary Table 1. The calculated IC_{50} values. Data are presented as mean values ± SD.

Drugs or nanoparticles	IC_{50} values from the 48 h MTS assay (μM)					
	HCT116	PANC-1	BXPC-3	IMR-90	NIH/3T3	Bone marrow
Lys05	8.0 ± 0.7	10.8 ± 0.9	11.1 ± 1.7	15.5 ± 2.5	12.6 ± 0.9	14.0 ± 1.1
BAQ12 NPs	1.6 ± 0.1	1.9 ± 0.4	2.6 ± 0.3	6.23 ± 0.8	6.5 ± 0.7	7.8 ± 0.8
BAQ13 NPs	1.6 ± 0.1	2.6 ± 0.3	3.1 ± 0.1	6.7 ± 0.5	7.0 ± 0.5	8.0 ± 0.8
Irinotecan	17.0 ± 1.0	--	--	--	--	--

Supplementary Table 2. The calculated pharmacokinetic parameters using Kinetica 5.0.

Nanoparticles	Cmax (ng mL^{-1})	AUC_{0-48}	$T_{1/2}$ (h)
Free DiD	8.6	34.9	0.8
BAQ12 NPs@DiD	10.6	124.7	12.4
BAQ13 NPs@DiD	11.0	182.0	15.9

Supplementary Table 3. Median survival of mice bearing HT29 tumours. n = 6 mice per group.

Groups	Median Survival (day)
Vehicle	21
Lys05	28
BAQ12 NPs	34
BAQ13 NPs (low)	36
BAQ13 NPs (high)	48
Irinotecan	36

Supplementary Table 4 Primers and sequences used in RT-PCR analysis.

Genes	Primers
BAD	5'-CCCAGAGTTTGAGCCGAGTG-3' (Forward)
	5'-CCCATCCCTTCGTCGTCCT-3' (Reverse)
BAX	5'-CCCGAGAGGTCTTTTTCCGAG-3' (Forward)
	5'-CCAGCCCATGATGGTTCTGAT-3' (Reverse)
BAK1	5'-GAGAGCCTGCCCTGCCCTCT-3' (Forward)
	5'-CCACCCAGCCACCCCTCTGT-3' (Reverse)
BIM	5'-GGCAAAGCAACCTTCTGATG-3' (Forward)
	5'-TAACCATTCGTGGGTGGTCT-3' (Reverse)
PUMA	5'-GACCTCAACGCACAGTACGAG-3' (Forward)
	5'-AGGAGTCCCATGATGAGATTGT-3' (Reverse)
ATP6AP1	5'-CAGCGACTTGCAGCTCTCTAC-3' (Forward)
	5'-TGAAATCCTCAATGCTCAGCTTG-3' (Reverse)
ATP6V0D1	5'-TTCCCGGAGCTTTACTTTAACG-3' (Forward)
	5'-CAAGTCCTCTAGCGTCTCGC-3' (Reverse)
ATP6V1E1	5'-AACATAGAGAAAGGTCGGCTTG-3' (Forward)
	5'-GACTTTGAGTCTCGCTTGATTCA-3' (Reverse)
ATP6V0E1	5'-GTCCTAACCGGGGAGTTATCA-3' (Forward)
	5'-AAAGAGAGGGTTGAGTTGGGC-3' (Reverse)
LAMP2	5'-GAAAATGCCACTTGCCTTTATGC-3' (Forward)
	5'-AGGAAAAGCCAGGTCCGAAC-3' (Reverse)
LAMP3/CD63	5'-CAGTGGTCATCATCGCAGTG-3' (Forward)
	5'-ATCGAAGCAGTGTGGTTGTTT (Reverse)
SQSTM1/p62	5'-GACTACGACTTGTGTAGCGTC-3' (Forward)
	5'-AGTGTCCGTGTTTCACCTTCC-3' (Reverse)
ATG2A	5'-TGTCCCTGTAGCCATGTTCG-3' (Forward)
	5'-TCAGGATCTCCGTGTACTCAG-3' (Reverse)
CLCN5	5'-ATAGGCACCGAGAGATTACCAA-3' (Forward)
	5'-CTAACGAACCTGATAAAAGCCCA-3' (Reverse)
CLCN6	5'-TCTCCTTACGGAAGATCCAGTT-3' (Forward)
	5'-AAGGTGGCAGACATGGAACAA-3' (Reverse)
CLCN7	5'-CCACGTTCACCCTGAATTTTGT-3' (Forward)
	5'-AAACCTTCCGAAGTTGATGAGG-3' (Reverse)
GAPDH	5'-TGTGGGCATCAATGGATTTGG-3' (Forward)
	5'-ACACCATGTATTCCGGGTCAAT-3' (Reverse)

Chemical structure characterization.

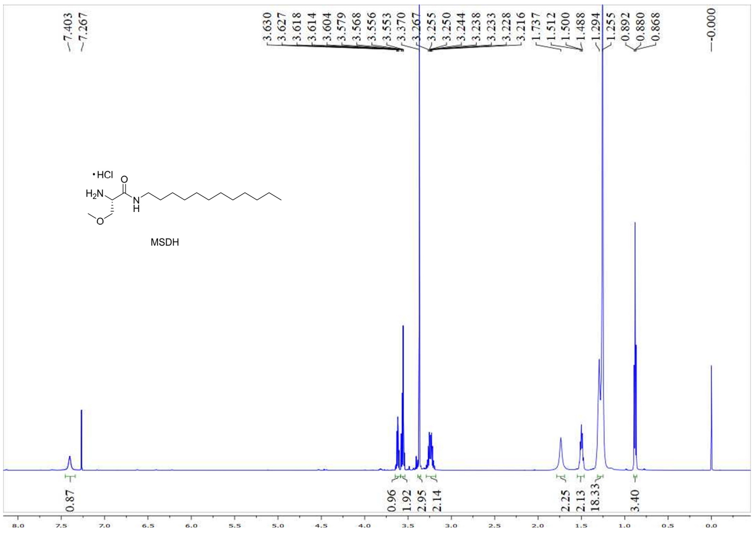

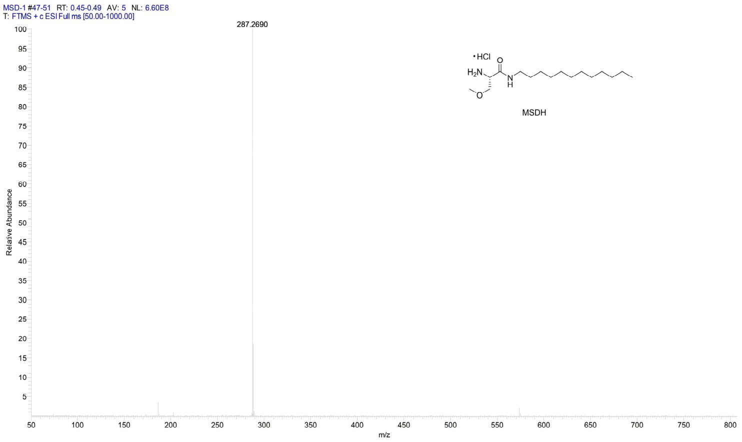

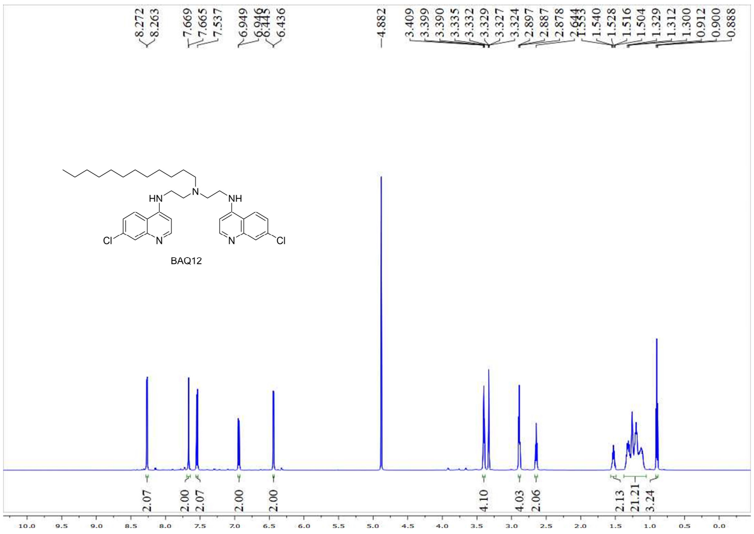
HN
N
NH
Cl
N
N
Cl
BAQ12

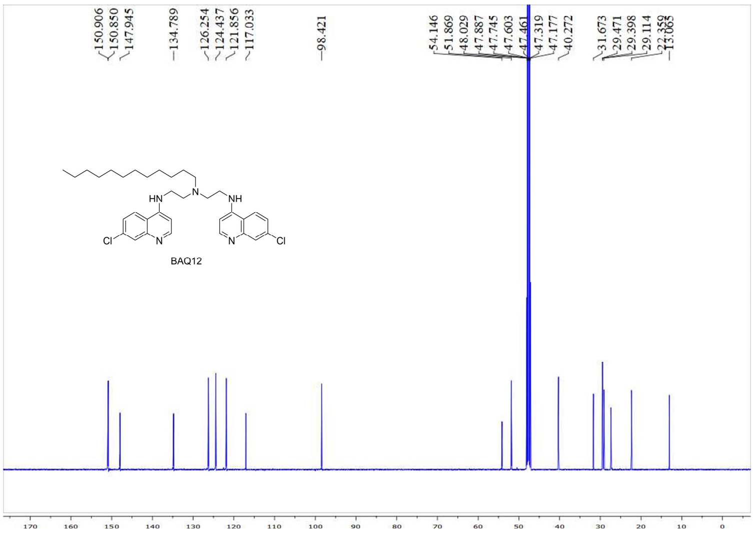
HN
N
NH
Cl
N
N
Cl
BAQ12

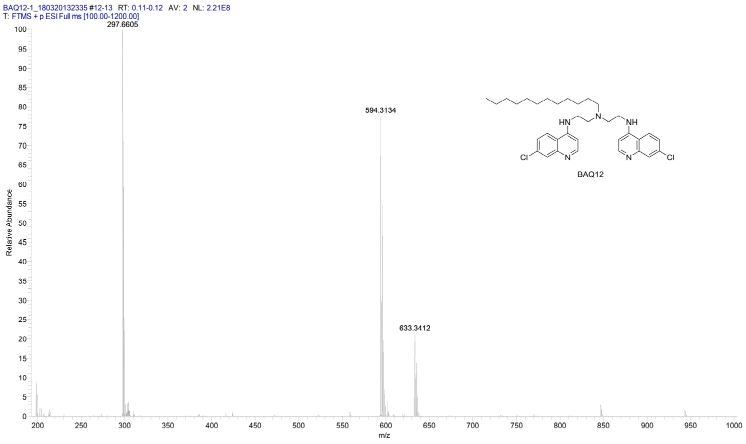
BAQ12-1_180320132335 #12-13 RT: 0.11-0.12 AV: 2 NL: 2.21E8
T: FTMS + p ESI Full ms [100.00-1200.00]
297.6605
594.3134
633.3412
Relative Abundance
m/z
HN
N
NH
Cl
N
N
Cl
BAQ12

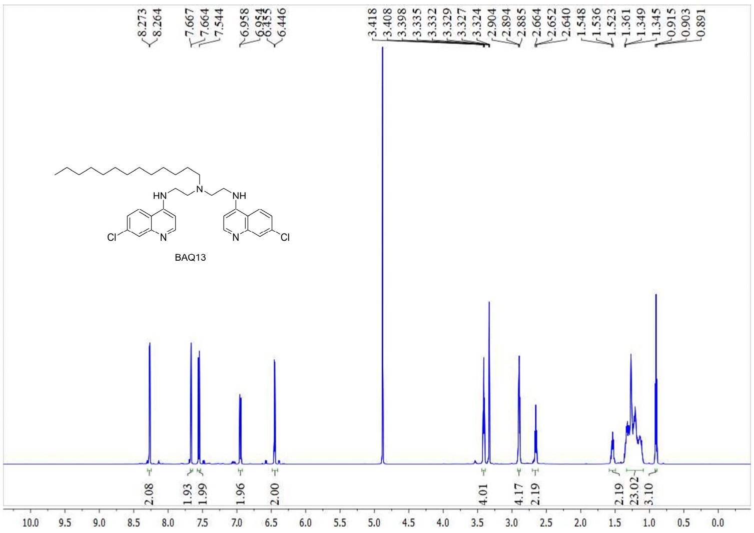
BAQ13

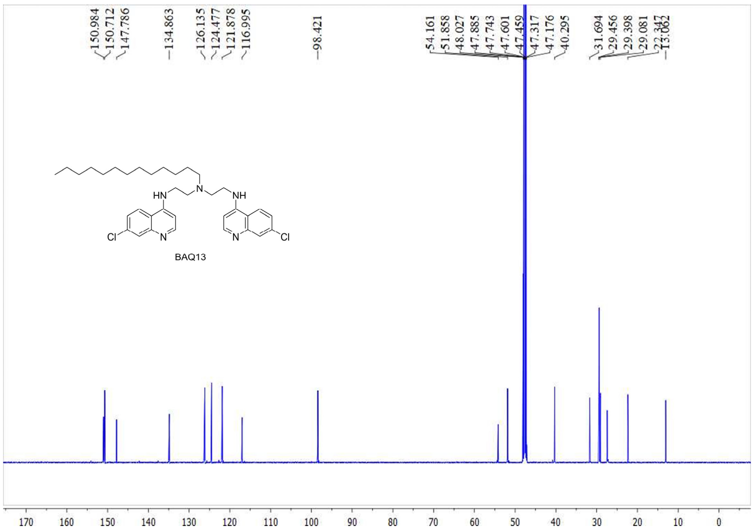
BAQ13

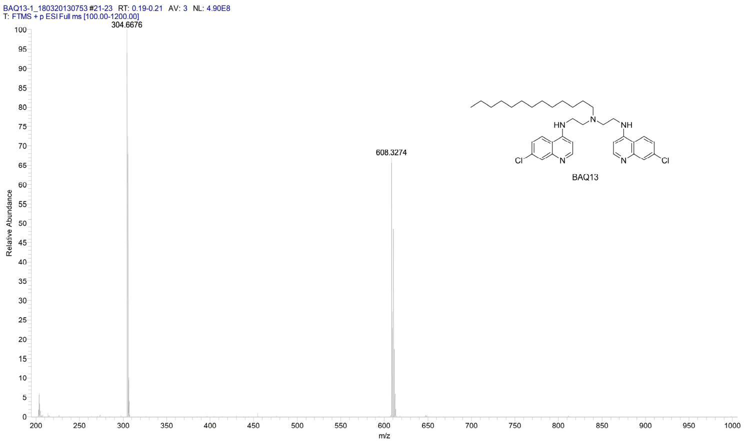
BAQ13-1_180320130753 #21-23 RT: 0.19-0.21 AV: 3 NL: 4.90E8
T: FTMS + p ESI Full ms [100.00-1200.00]
304.6676
608.3274
Relative Abundance
m/z
BAQ13

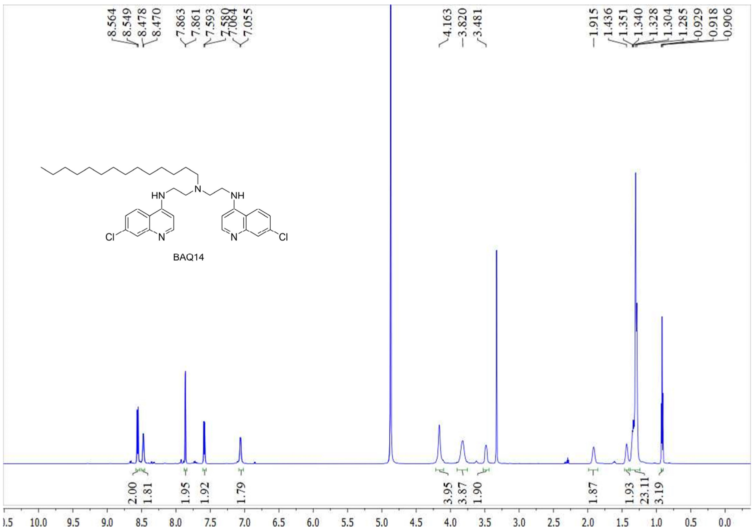
8.564
8.549
8.478
8.470
7.863
7.861
7.593
7.580
7.064
7.055
4.163
3.820
3.481
1.915
1.436
1.351
1.340
1.328
1.304
1.285
0.929
0.918
0.906
HN
N
NH
Cl
N
N
Cl
BAQ14
2.00
1.81
1.95
1.92
1.79
3.95
3.87
1.90
1.87
1.93
23.11
3.19
10.0 9.5 9.0 8.5 8.0 7.5 7.0 6.5 6.0 5.5 5.0 4.5 4.0 3.5 3.0 2.5 2.0 1.5 1.0 0.5 0.0

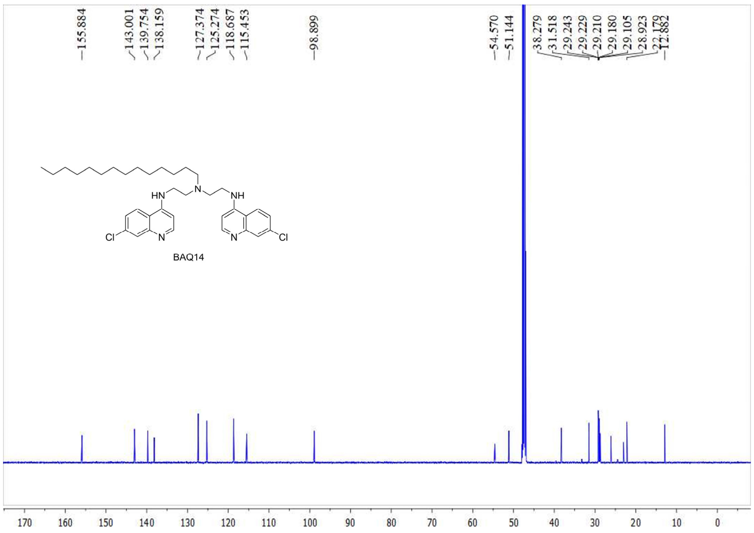
155.884
143.001
139.754
138.159
127.374
125.274
118.687
115.453
98.899
54.570
51.144
38.279
31.518
29.243
29.229
29.210
29.180
29.105
28.923
12.882
HN
N
NH
Cl
N
N
Cl
BAQ14
170 160 150 140 130 120 110 100 90 80 70 60 50 40 30 20 10 0

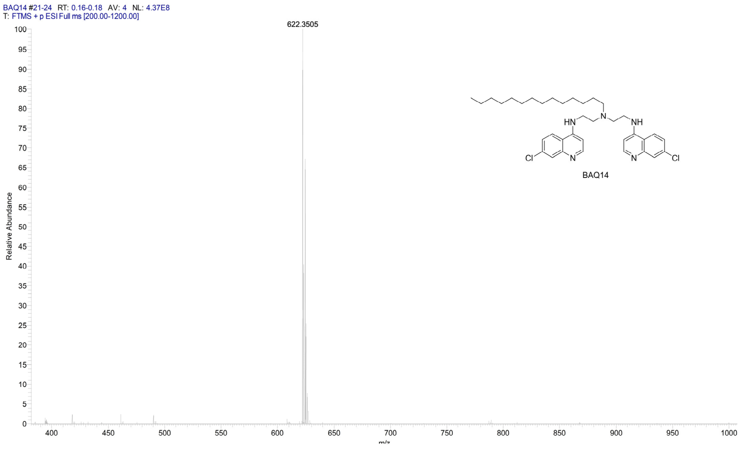
BAQ14 #21-24 RT: 0.16-0.18 AV: 4 NL: 4.37E8
T: FTMS + p ESI Full ms [200.00-1200.00]
622.3505
Relative Abundance
100 95 90 85 80 75 70 65 60 55 50 45 40 35 30 25 20 15 10 5 0
400 450 500 550 600 650 700 750 800 850 900 950 1000
HN
N
NH
Cl
N
N
Cl
BAQ14

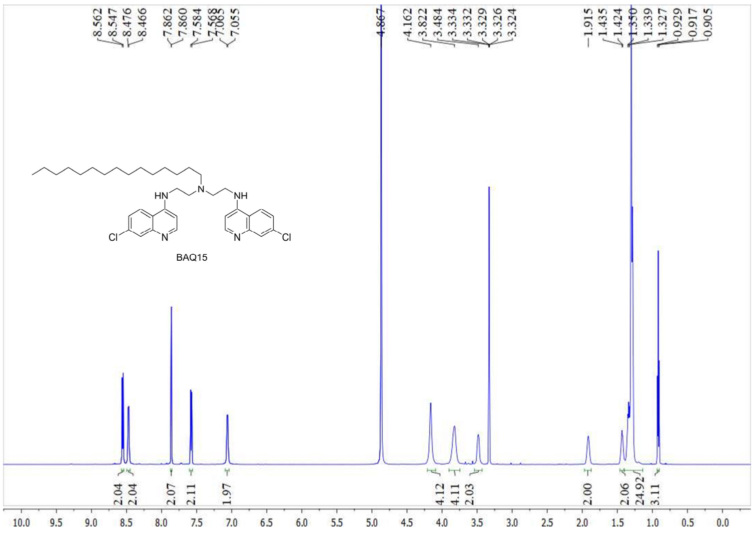
HN
N
NH
Cl
N
N
Cl
BAQ15

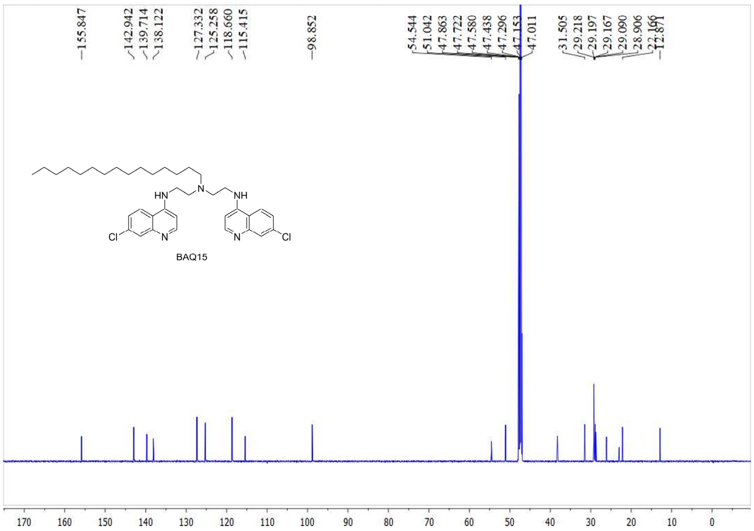
HN
N
NH
Cl
N
N
Cl
BAQ15

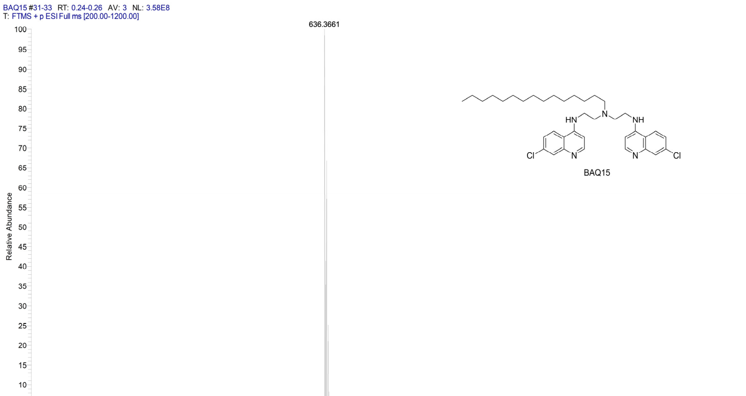
BAQ15 #31-33 RT: 0.24-0.26 AV: 3 NL: 3.58E8
T: FTMS + p ESI Full ms [200.00-1200.00]
636.3661
Relative Abundance
HN
N
NH
Cl
N
N
Cl
BAQ15

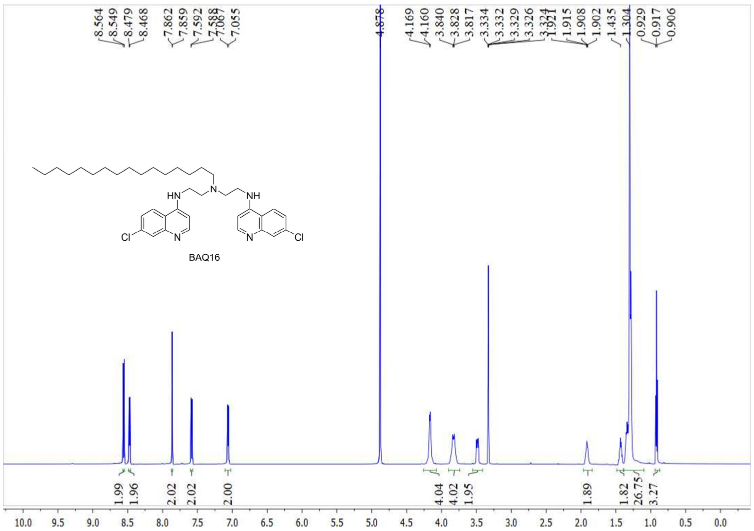
BAQ16
HN
NH
N
Cl
Cl
8.564
8.549
8.479
8.468
7.862
7.859
7.592
7.588
7.067
7.055
4.878
4.169
4.160
3.840
3.828
3.817
3.334
3.332
3.329
3.326
1.921
1.915
1.908
1.902
1.435
1.304
0.929
0.917
0.906
1.99
1.96
2.02
2.02
2.00
4.04
4.02
1.95
1.89
1.82
26.75
3.27

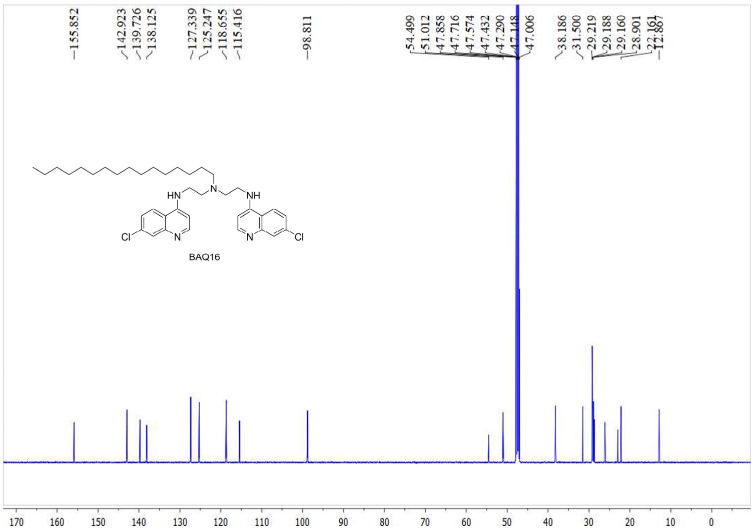
BAQ16
155.852
142.923
139.726
138.125
127.339
125.247
118.655
115.416
98.811
54.499
51.012
47.858
47.716
47.574
47.432
47.290
47.148
47.006
38.186
31.500
29.219
29.188
29.160
28.901
22.161
12.897

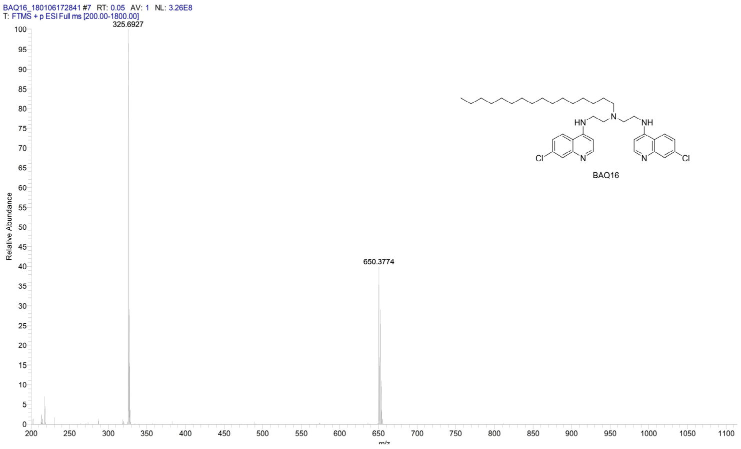
BAQ16_180106172841 #7 RT: 0.05 AV: 1 NL: 3.26E8
T: FTMS + p ESI Full ms [200.00-1800.00]
325.6927
650.3774
Relative Abundance
m/z
BAQ16

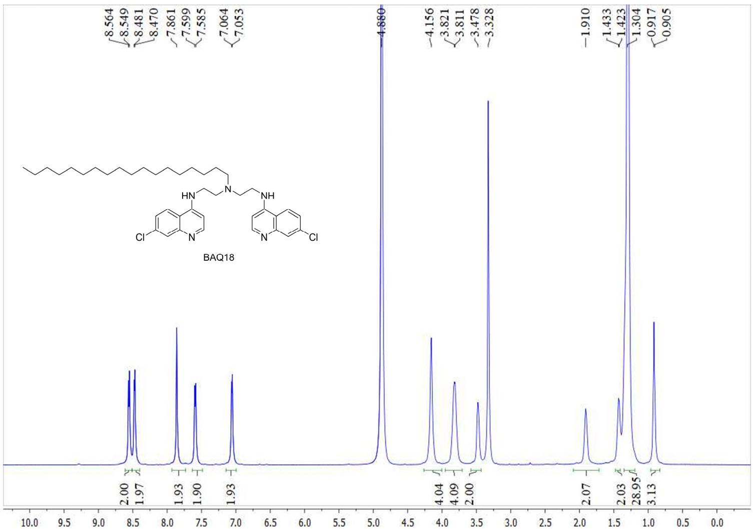

BAQ18

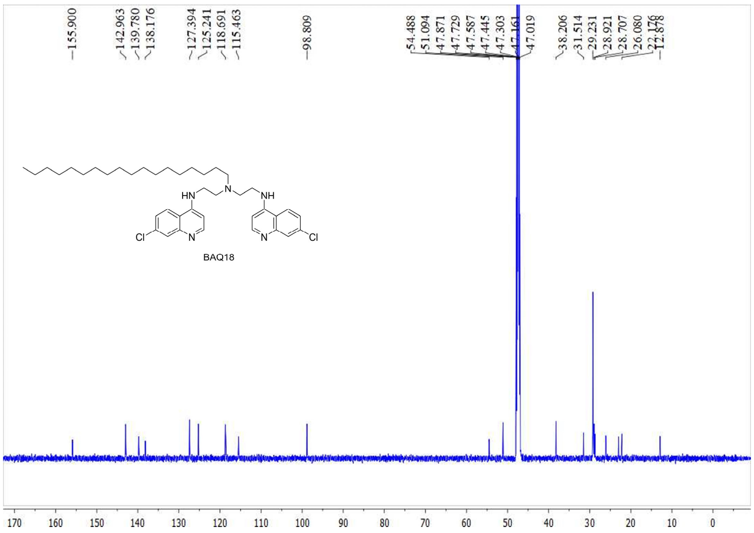

BAQ18

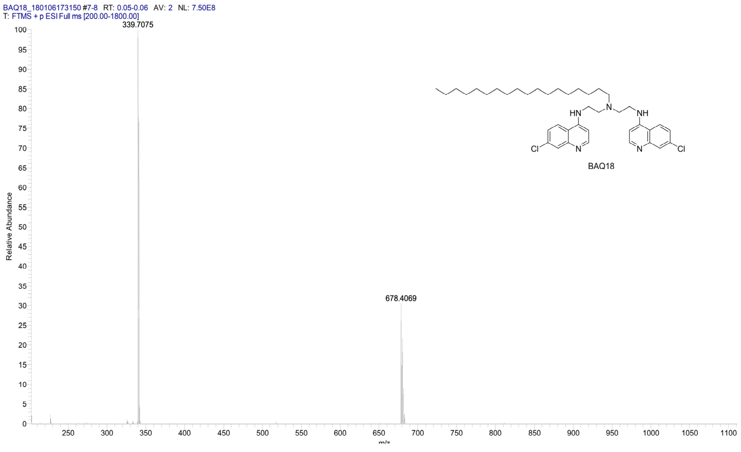

BAQ18_180106173150 #7-8 RT: 0.05-0.06 AV: 2 NL: 7.50E8
T: FTMS + p ESI Full ms [200.00-1800.00]
339.7075
678.4069
Relative Abundance
BAQ18
