# Supplementary material for: Pharmacophore hybridisation and nanoscale assembly to discover self-delivering lysosomotropic new-chemical entities for cancer therapy
Source: Nat Commun. 2020 Sep 15;11:4615. doi: 10.1038/s41467-020-18399-4 (PMC7493904; doi:10.1038/s41467-020-18399-4)
Supplement: Supplementary file 3 — Reporting Summary [file 41467_2020_18399_MOESM3_ESM.pdf]

nature research

Corresponding author(s): Yuanpei Li

Last updated by author(s): Jul 19, 2020

Reporting Summary

Nature Research wishes to improve the reproducibility of the work that we publish. This form provides structure for consistency and transparency in reporting. For further information on Nature Research policies, see Authors & Referees and the Editorial Policy Checklist .

Statistics

For all statistical analyses, confirm that the following items are present in the figure legend, table legend, main text, or Methods section.

n/a	Confirmed	
☐	☒	The exact sample size (*n*) for each experimental group/condition, given as a discrete number and unit of measurement
☐	☒	A statement on whether measurements were taken from distinct samples or whether the same sample was measured repeatedly
☐	☒	The statistical test(s) used AND whether they are one- or two-sided<br>*Only common tests should be described solely by name; describe more complex techniques in the Methods section.*
☐	☒	A description of all covariates tested
☐	☒	A description of any assumptions or corrections, such as tests of normality and adjustment for multiple comparisons
☐	☒	A full description of the statistical parameters including central tendency (e.g. means) or other basic estimates (e.g. regression coefficient) AND variation (e.g. standard deviation) or associated estimates of uncertainty (e.g. confidence intervals)
☐	☒	For null hypothesis testing, the test statistic (e.g. *F*, *t*, *r*) with confidence intervals, effect sizes, degrees of freedom and *P* value noted<br>*Give P values as exact values whenever suitable.*
☒	☐	For Bayesian analysis, information on the choice of priors and Markov chain Monte Carlo settings
☐	☒	For hierarchical and complex designs, identification of the appropriate level for tests and full reporting of outcomes
☐	☒	Estimates of effect sizes (e.g. Cohen's *d*, Pearson's *r*), indicating how they were calculated

Our web collection on statistics for biologists contains articles on many of the points above.

Software and code

Policy information about availability of computer code

Data collection	NMR spectrometer (Bruker 600 MHz, German); LTQ-Orbitrap XL Hybrid ion trap mass spectrometer (Thermo Fisher, USA); Zetasizer Nano ZS (Malvern Instruments, UK); Talos L120C TEM (FEI, USA); UV-vis photospectrometer (Shimadzu UV-1800, Japan); Fluorescence photospectrometer Shimadzu RF6000, Japan); Fluorescence microscope (Olympus, Japan); LSM800 confocal microscope (Carl Zeiss, Oberkochen, Germany); SpectraMax M2 microplate reader (Molecular Devices, CA, USA); ChemiDocTM MP imaging system (Bio-Rad, USA); BD FACSCanto II flow cytometer and BD FACSAria II Cell Sorter (BD Biosciences, NJ, USA).
Data analysis	Bar graphs and the relevant statistics were analyzed by GraphPad Prism 7.0; Cell images were analyzed by Zen 2.3 and ImageJ 1.51s; Western blot images and animal images were analyzed by Image lab 6.0; Drug combination data were analyzed by Combenefit 2.02; Flow cytometry data were analyzed by FlowJo 7.6.1. RNA-seq: The Differentially expressed genes (DEGs) were collected for the signaling pathways enrichment by Funrich software 3.1.3. Lipidomics: Untargeted lipidomic data processing was performed using MS-DIAL 3.70 and Normalized peak heights were then submitted to R 3.5.1 for statistical analysis. The gene sets were from MSigDB database (Broad Institute). GSEA was performed using GSEA version 3.0 in KEGG gene sets category online.

For manuscripts utilizing custom algorithms or software that are central to the research but not yet described in published literature, software must be made available to editors/reviewers. We strongly encourage code deposition in a community repository (e.g. GitHub). See the Nature Research guidelines for submitting code & software for further information.

Data

Policy information about availability of data

All manuscripts must include a data availability statement. This statement should provide the following information, where applicable:

- Accession codes, unique identifiers, or web links for publicly available datasets
- A list of figures that have associated raw data
- A description of any restrictions on data availability

The RNA-seq data have been deposited in the Gene Expression Omnibus (GEO) database under the accession code GSE154323 [https://www.ncbi.nlm.nih.gov/geo/query/acc.cgi?acc=GSE154323]. The source data underlying Figs 2a-g, 3e-g, i, k, 4d-g, i-j, 5a, c-f, i, 6c, e, g, i, l and Supplementary Figs 2a, c, 3a-b, d-f, 4b, e, 5c-f, 6,

7b-f, 8b-e, 9a-c, 11c-d are provided as a Source Data file. All the other data supporting the findings of this study are available within the article and its supplementary information files and from the corresponding author upon reasonable request. A reporting summary for this article is available as a Supplementary Information file.

Field-specific reporting

Please select the one below that is the best fit for your research. If you are not sure, read the appropriate sections before making your selection.

- [x] Life sciences
- [ ] Behavioural & social sciences
- [ ] Ecological, evolutionary & environmental sciences

For a reference copy of the document with all sections, see nature.com/documents/nr-reporting-summary-flat.pdf

Life sciences study design

All studies must disclose on these points even when the disclosure is negative.

Sample size	No statistical tests were used to determine sample size prior to starting study. Sample sizes were deemed to be sufficient based on size of effects seen and reaching statistical significance based on the available samples. Three independent nanoparticle samples were used for characterization of nanoparticle. At least three independent cell samples per group were used for cell-based experiments. For in vivo experiments, there were at least three mice per group included in the biodistribution study, at least four mice per group in toxicity study, and at least 5 mice and 6-12 tumors per group in the treatment study.
Data exclusions	No data was excluded for analysis.
Replication	All the experiments were performed three times independently. All the attempts at replication were successful with the similar results and expected range of standard deviation.
Randomization	Samples of nanoparticles, cells and mice, were allocated to groups randomly prior to treatment. Different cell passages (<15) were used for each biological replicate.
Blinding	No blinding was performed. The data and results are not subjective and quantitative data are provided when possible, which removes the need for blinding.

Reporting for specific materials, systems and methods

We require information from authors about some types of materials, experimental systems and methods used in many studies. Here, indicate whether each material, system or method listed is relevant to your study. If you are not sure if a list item applies to your research, read the appropriate section before selecting a response.

Materials & experimental systems

n/a	Involved in the study	
[ ]	[x]	Antibodies
[ ]	[x]	Eukaryotic cell lines
[x]	[ ]	Palaeontology
[ ]	[x]	Animals and other organisms
[ ]	[x]	Human research participants
[x]	[ ]	Clinical data

Methods

n/a	Involved in the study	
[x]	[ ]	ChIP-seq
[ ]	[x]	Flow cytometry
[x]	[ ]	MRI-based neuroimaging

Antibodies

Antibodies used	LC3B antibody (Cell Signaling, Catalog: #2775, 1:1000); SQSTM1/p62 mAb (Cell Signaling, Catalog: #39749, 1:1000); β-actin mAb (Cell Signaling, Catalog: #4970, 1:1000); Pacific Blue anti-CD44 antibody (BioLegend, Catalog: #338823, 5 µl per million cells in 100 µl staining volume); APC anti-CD326 (EpCAM) antibody (BioLegend, Catalog: #324207, 5 µl per million cells in 100 µl staining volume); PE/Cy7 anti-CD24 antibody (BioLegend, Catalog: #311119, 5 µl per million cells in 100 µl staining volume).
Validation	Each antibody was commercially validated for the application used by the manufacturer.<br>(1) LC3B Rabbit Antibody (Cat#2775, Cell Signaling):<br>https://www.cellsignal.com/products/primary-antibodies/lc3b-antibody/2775<br>-Antibody was validated for western blotting by Cell Signaling. "Western blot analysis of extracts from HeLa cells, mock transfected or transfected with rat LC3B, and from HT-1080 and A20 cells, untreated or chloroquine-treated (50 µM, overnight), using LC3B Antibody."<br>-Published 1131 times.<br>(2) β-Actin Rabbit mAb (Cat#4970, Cell Signaling):<br>https://www.cellsignal.com/products/primary-antibodies/b-actin-13e5-rabbit-mab/4970<br>-Antibody was validated for western blotting by Cell Signaling. "Western blot analysis of cell extracts from various cell lines using

beta-Actin (13E5) Rabbit mAb".
-Published 2258 times.
(3) SQSTM1/p62 (D1Q5S) Rabbit mAb (Cat#39749, Cell Signaling):
https://www.cellsignal.com/products/primary-antibodies/sqstm1-p62-d1q5s-rabbit-mab/39749
-Antibody was validated for western blotting by Cell Signaling. "Western blot analysis of extracts from various cell lines using SQSTM1/p62 (D1Q5S) Rabbit mAb (upper) or β-Actin (D6A8) Rabbit mAb #8457 (lower)."
-Published 34 times
(4) Pacific Blue™ anti-human CD44 Antibody (Cat#338823, Biolegend)
https://www.biolegend.com/en-us/products/pacific-blue-anti-human-cd44-antibody-15263
-Quality was tested for flow cytometry by Biolegend.
(5) APC anti-human CD326 (EpCAM) Antibody (Cat#324207, Biolegend)
https://www.biolegend.com/en-us/products/apc-anti-human-cd326-epcam-antibody-3758
-Quality was tested for flow cytometry by Biolegend.
-Published 24 times
(6) PE/Cyanine7 anti-human CD24 Antibody (Cat#311119, Biolegend)
https://www.biolegend.com/en-us/products/pe-cyanine7-anti-human-cd24-antibody-6126
-Quality was tested for flow cytometry by Biolegend.
-Published 10 times

Eukaryotic cell lines

Policy information about cell lines

Cell line source(s)	The human pancreatic cancer cell lines (MIA PaCa-2, BXPC3 and PANC-1) were originally purchased from ATCC and were kindly provided by Dr. Shiro Urayama's Lab, HT29, HCT116, H460, MCF7, NIH/3T3 and IMR-90 cell lines were purchased from ATCC. Bone marrow cells were collected from the leg bone marrows of FVB/N mice. The pancreatic cancer stem cells were isolated from the pancreatic patient tissue, which was also donated by Dr. Shiro Urayama's Lab.
Authentication	Cell line authentication was performed by short tandem repeat DNA profiling.
Mycoplasma contamination	All the cell lines have been tested for mycoplasma contamination routinely. No mycoplasma contamination was found.
Commonly misidentified lines (See ICLAC register)	No commonly misidentified lines were used in this study.

Animals and other organisms

Policy information about studies involving animals; ARRIVE guidelines recommended for reporting animal research

Laboratory animals	The female mice (4-6 week), including BALB/c nude mice (Envigo), NRG mice (Jackson Laboratory) and FVB/N mice (Charles River), were used in the study.
Wild animals	There were no wild animals involved in the study.
Field-collected samples	This study did not involve samples collected from the fields.
Ethics oversight	All animal experiments were in accordance with the protocol #20265, which was approved by the Animal Use and Care Administrative Advisory Committee at the University of California, Davis.

Note that full information on the approval of the study protocol must also be provided in the manuscript.

Human research participants

Policy information about studies involving human research participants

Population characteristics	Cases included in the Study consists of any adult patients who were referred to pancreatic endoscopic study at our medical center (excluded includes any protected population for research participation as prescribed by human research protection guideline). Consent obtained from the patients for remnant biospecimens utilization, obtained prior to the procedure, for cellular and molecular characterization.
Recruitment	All adult patients who were referred to pancreatic endoscopic procedure were asked to be enrolled in the Pancreas Registry/ Biobank study for cellular and molecular characterization – banking of the small biospecimen for later or concurrent utilization for the characterization of the cells and molecular signals. A potential bias of cellular characterization arises as the data described are from the successfully propagating cancerous cells only, as opposed to all of the cells being harvested within the partially sampled zone of the whole tumor. Overall tendency, however, is that potential consequence of the added selection pressure from the cell processing would enhance the successfully propagating cells as more resistant, or capable of withstanding harsher environmental conditions.
Ethics oversight	Patient consent was obtained for the use of "Remnant Clinical Biospecimens" in accordance with the Institutional Review Board (UC Davis IRB Protocol #244896).

Note that full information on the approval of the study protocol must also be provided in the manuscript.

Flow Cytometry

Plots

Confirm that:

- [x] The axis labels state the marker and fluorochrome used (e.g. CD4-FITC).
- [x] The axis scales are clearly visible. Include numbers along axes only for bottom left plot of group (a 'group' is an analysis of identical markers).
- [x] All plots are contour plots with outliers or pseudocolor plots.
- [x] A numerical value for number of cells or percentage (with statistics) is provided.

Methodology

Sample preparation	For apoptosis assay, cells that were treatments as indicated for 24h, followed by the staining with a FITC-Annexin V/PI Apoptosis kit (AnaSpec) according to the manufacturer's instruction. The procedures for cancer stem cell population isolation are (1) Harvest cells at appropriate confluence with trypsinization; (2) Spin the cells with 1000 rpm for 5 min; (3) Discard the supernatant and resuspend the cells with staining buffer (1% BSA or human serum albumin (HSA)); (4) Filter the cells with cell strainer (70 μm) and count the cell number; (5) Spin the cells again with 1000 rpm for 5 min; (6) Discard the supernatant and resuspend the cell pellet with an appropriate; (7) Calculate the volume staining buffer is calculated based on staining 1 million cells in 100 μL, and stain the cells as the following: (a) The cells without staining (around 200,000-300,000 cells) in 500 μL of staining buffer; (b) The cells are only stained with an antibody with color A (for example CD24) in 100 μL with 300,000 cells; (c) The cells are only stained with an antibody with color B (for example CD44) in 100 μL with 300,000 cells; (d) The cells are only stained an antibody with color C (for example EpCAM) in 100 μL with 300,000 cells; e) The major population of cells are stained with three antibodies in appropriate volume (not exceed 500 μL, 1 million cells in 100 μL); multiple staining tubes may be applied if the total cells are over 5 millions; (f) The amounts of antibodies and the incubation time/temperature are employed per suggestion from manufacture; (8) Wash the cells with staining buffer: no washing for 7a, wash the cells with 3 mL of staining buffer for 7b, c, and d, and wash the cells with 9 mL of staining buffer if the staining volume is 500 μL; (9) Spin the cells with 1000 rpm for 5 min except the cells from 7a; (10) Resuspend the cells with 500 μL of staining buffer for staining with single dye, and resuspend the cells for sorting at 10 million cells per mL; (11) Add PI into the cell suspension during the sorting, and follow the instruction from the operator of the sorter; (12) Collect the cells with collection buffer (50% FBS + 50% HAM medium); (13) Spin the cells after sorting with 1200 rpm for 5 min; (14) Discard the supernatant and re-suspend the cells with small volume of HAM medium; (15) Seed the cells into each well with around 50,000 cells under the condition with the feeder cells which is prepared one day before sorting.
Instrument	BD FACSCanto I and BD FACSAria II Cell Sorter
Software	Flow cytometry data was collected with BD FACS Diva software and analyzed with FlowJo 10.
Cell population abundance	Single positive (CD326+) cells were about 77% of the whole population; Double positive (CD326+ CD24+) cells were around 50% of the single positive population; Triple positive (CD326+, CD24+, CD44+) cells were about 30% of the double positive population and about 10% of the whole population.
Gating strategy	All the cell populations were chosen after excluding cell debris for apoptosis assay. The cell population in early apoptosis was indentified as FITC-Annexin V+/PI- cells, and in late apoptosis was indentified as FITC-Annexin V+/PI+ cells. For cell sorting, after excluding the PI+ positive dead cells, negative control (unstained with antibody-conjugated dye) and dye-positive cells were used to establish gates. Gates were drawn to collect cells expressing all the three dyes. See the provided examples for gates used.

- [x] Tick this box to confirm that a figure exemplifying the gating strategy is provided in the Supplementary Information.
